# Potential Application of Cardiac Computed Tomography for Early Detection of Coronary Atherosclerosis: From Calcium Score to Advanced Atherosclerosis Analysis

**DOI:** 10.3390/jcm10030521

**Published:** 2021-02-01

**Authors:** Edoardo Conte, Saima Mushtaq, Davide Marchetti, Vincenzo Mallia, Marta Belmonte, Eleonora Melotti, Gianluca Pontone, Mauro Pepi, Daniele Andreini

**Affiliations:** 1 Department of Cardiovascular Imaging, Centro Cardiologico Monzino, IRCCS, University of Milan, 20100 Milan, Italy; edoardo.conte@ccfm.it (E.C.); saima.mushtaq@ccfm.it (S.M.); davide.marchetti@unimi.it (D.M.); vincenzo.mallia@unimi.it (V.M.); belmontemarta1@gmail.com (M.B.); eleonora.melotti@unimi.it (E.M.); gianluca.pontone@ccfm.it (G.P.); mauro.pepi@ccfm.it (M.P.); 2 Department of Clinical Sciences and Community Health, Cardiovascular Section, University of Milan, 20100 Milan, Italy

**Keywords:** primary prevention, coronary artery calcium score, coronary computed tomography angiography, high-risk plaque features, quantitative coronary plaque analysis

## Abstract

In the present article, an overview of advanced analysis of coronary atherosclerosis by coronary computed tomography angiography (CCTA) is provided, focusing on the potential application of this technique in a primary prevention setting. Coronary artery calcium score (CACS) has a well-demonstrated prognostic value even in a primary prevention setting; however, fibro-fatty, high-risk coronary plaque may be missed by this tool. On the contrary, even if not recommended for primary prevention in the general population, CCTA may enable early high-risk atherosclerosis detection, and specific subgroups of patients may benefit from its application. However, further studies are needed to determine the possible use of CCTA in a primary prevention setting.

## 1. Introduction

The prevalence of traditional risk factors and overall cardiovascular mortality have been constantly decreasing in the last two decades, as recently reported by the European Society of Cardiology (ESC) [1]; however, a mild increase in cardiovascular disease (CVD) incidence between 1990 and 2015 has been registered in both women and men [1].

According to primary prevention ESC guidelines, systematic cardiovascular risk assessment may be considered in men >40 years old and in women >50 years old, and adoption of risk estimation systems (i.e., SCORE) is recommended [2]. However, the presence of risk modifiers, which include computed tomography CT coronary artery calcium score (CACS) as per ESC guidelines, could reclassify patients otherwise considered at low risk.

In the last few decades, coronary computed tomography angiography (CCTA) gained wide diffusion in the clinical field for noninvasive evaluation of coronary artery disease. This important role is supported by an extensive amount of literature demonstrating a good diagnostic performance vs. invasive coronary angiography [3,4], together with a prognostic value of CCTA that enables identification of patients at higher risk of future major cardiovascular events (MACE) among patients with stable or suspected coronary artery disease (CAD) [5,6,7]. Recently published NICE/UK guidelines [8] suggested CCTA as a first-step test in patients with suspected CAD, but it must be underlined that the use of CCTA in asymptomatic patients without known cardiovascular disease is still uncertain and only measurement of CACS is considered appropriate in a primary prevention setting according to most recent guidelines [9,10,11].

Recent data may suggest a potential role of CCTA in the setting of primary prevention, thanks to the capability of this technique to provide an extensive evaluation of coronary atherosclerosis, improving early and accurate identification of patients at a higher risk of future coronary events, even if asymptomatic and without known cardiovascular disease.

## 2. Noninvasive Coronary Atherosclerosis Assessment by CT

### 2.1. Cardiac CT: Technological Background

When compared to a traditional thoracic CT scan, the evaluation of cardiac structures needs the intrinsic heart movement and related artifacts to be considered and incorporated. In the last two decades, technological advances, especially with the wide clinical application of the multidetector CT scan, allowed the advent of ECG-gated cardiac CT. More precisely, thanks to ECG-gated CT, only the diastolic phase of the cardiac cycle is selected and acquired, enabling the obtainment of images of coronary arteries despite their physiological movement. Coronary calcium burden is evaluated without the need for contrast medium, and the prognostic value of the coronary artery calcium score (CACS) has been widely validated. With the adjunct of intravenous contrast medium, coronary artery lumen can be noninvasively evaluated with ECG-gated contrast CT scans acquired at the early angiographic phase, paving the way to the first noninvasive tool for coronary anatomy evaluation. This type of acquisition was performed with a non-negligible increase of radiation dose (especially when a 16-slice CT was used) that has been progressively reduced to less than 3–5 mSv after the advance of whole-heart coverage CT scans (i.e., 256-slice CT).

### 2.2. Coronary Artery Calcium Score (CACS): Noncontrast CT

**Figure 1:**
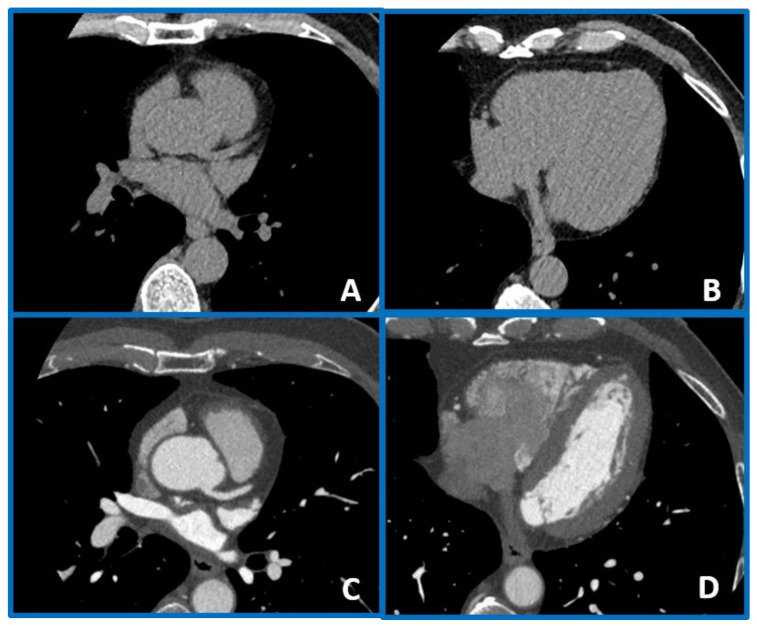
A case example of a 50-year-old man with arterial hypertension and noncardiac chest pain in which a coronary artery calcium score (CACS) value of 0 (panels (**A**,**B**)) was associated with no coronary plaque at coronary computed tomography angiography (CCTA) (panels (**C**,**D**)). In this case, CCTA evaluation did not add any information on top of CACS.

**Figure 2:**
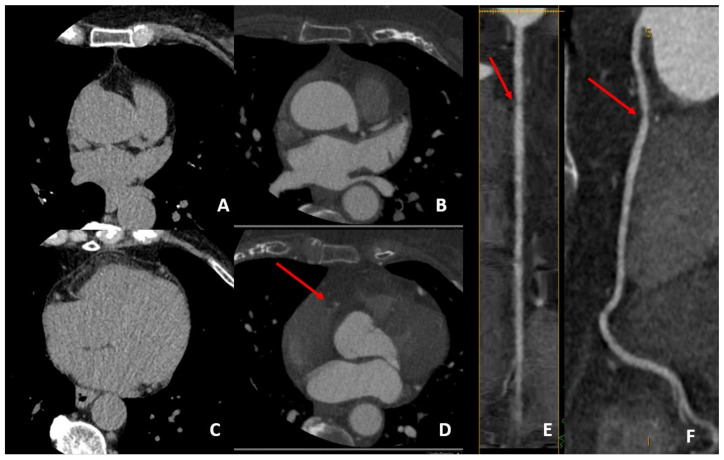
A case example of a middle-aged man with a family history of coronary artery disease (CAD) and noncardiac chest pain who was found to have a CACS value of 0 (panels (**A**,**B**)), but with identification at CCTA of fibro-lipidic plaque on proximal RCA (panels (**C**,**D**) in axial view). This high risk (positive remodeling and low-attenuation plaque) causes a moderate stenosis (50% to 70% stenosis) that is well evidenced at curved analysis (red arrow on panels (**E**,**F**)). This may merit aggressive cardiovascular risk factor control with preventive therapy in order to reduce the future risk of cardiovascular events that may be underestimated by CACS alone.

**Figure 3:**
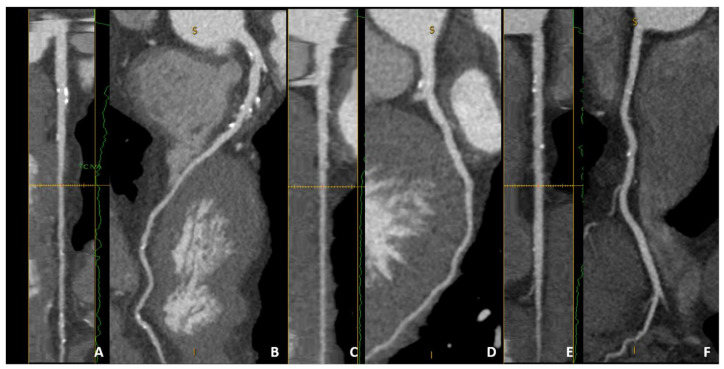
A case example of an asymptomatic 70-year-old female with multiple risk factors. Moderate CACS values (between 100 and 400) can be associated with diffuse but nonsignificant calcified plaque. Of note, no high-risk features or severe stenosis were identified on LAD (panels (**A**,**B**)), LCX (panels (**C**,**D**), or RCA (panels (**E**,**F**)). Of interest, both CACS and CCTA identify a low-to-moderate risk of future cardiovascular events despite multiple cardiovascular risk factors.

CACS can be considered the first approach to coronary atherosclerosis evaluation by cardiac CT beyond coronary lumen stenosis quantification. Performed on noncontrast cardiac CT images, coronary artery calcium (CAC) is defined as a hyperattenuated lesion of at least 1 mm^2^ above a threshold of 130 HU; a CAC score is then obtained by taking into consideration both the total area and the attenuation values of coronary atherosclerosis [12]. CACS evaluation has no immediate risk for the patient, but potential benefits must be weighed against the potential risk of exposure to ionizing radiation that should be kept as low as reasonably achievable [13]. The prognostic role of CACS both alone and in addition to traditional cardiovascular risk factors has been previously suggested. One of the first reports on the prognostic value of CACS in asymptomatic subjects was published in 2003 by Kondos et al., reporting that an elevated CACS was associated with an increased risk of cardiac events with a relative risk of 7.2 at a mean follow-up of 37 months [14]. Similarly, in another landmark paper from Budoff et al. published in 2007 including more than 25,000 asymptomatic subjects, identification of higher CACS values was associated with a higher risk of mortality at 6 ± 3 years of follow-up [15]. Subsequently, a study including more than 1800 consecutive asymptomatic patients suggested that CACS may significantly reclassify cardiovascular risk when compared to traditional risk factors alone [16], while a CONFIRM substudy suggested that the composite evaluation of risk factors and CACS reached a similar prognostic value of CCTA for all-cause mortality at a follow-up of 5.9 ± 1.2 years[17]. Moreover, in 2005, prediction of cardiovascular events (such as acute coronary syndromes and sudden cardiac death) was improved by electron beam CT-derived CACS in a population-based study involving 4903 asymptomatic middle-aged Caucasians, when compared with standard coronary risk factors at a follow-up of 3 ± 1.4 years [6]. More recently, results from the Multi-Ethnic Study of Atherosclerosis (MESA) suggested that individuals with no risk factors but a CACS >300 had an event rate 3.5 times higher in terms of myocardial infarction, resuscitated cardiac arrest, and cardiac death when compared to individuals with more than three risk factors but a CACS of 0 (10.9/1000 vs. 3.1/1000 person-years) at a follow-up of 7 ± 1 years; these results suggested that the presence of an elevated CACS among individuals without risk factors is associated with an elevated events rate, whereas the absence of coronary artery calcium appeared to be protective [18] (Figure 1). In 2017, the Society of Cardiovascular Computed Tomography defined as appropriate the use of CACS in selected asymptomatic patients [19], suggesting that a CACS >0 may support statin therapy beyond standard indication and a CACS > 100 may identify patients who may benefit from aspirin therapy [20]. Similar recommendations have been recently included in 2019 American College of Cardiology/American Heart Associtation (ACC/AHA) guidelines for primary prevention [21]. It should be underlined that CACS estimates the extension of calcified plaque, while fibro-lipidic coronary atherosclerosis could be underestimated in a low, but clinically significant, number of patients (Figure 2). In this regard, in 2016, Dedic A. et al. confirmed the excellent prognostic value of CACS of 0 vs. CACS 0–100 and CAC >400 in a cohort of 665 high-risk patients, but these authors reported that CCTA identified CAD in 38% of patients with a CACS of 0. These data may suggest that even patients with a very low CACS may have significant CAD, and CCTA could better discriminate patients at a higher risk of adverse coronary events [22,23]. Thus, even if CACS is a well-validated and important tool for patient stratification on a population level, some concerns have been raised, as patients with a very low CACS can have high-risk atherosclerosis, and among patients with an intermediate-to-high CACS, a wide range of coronary lumen stenosis could be identified (Figure 3).

### 2.3. Coronary Artery Evaluation by CCTA

CCTA represents an important novelty in the evaluation of suspected CAD, enabling the anatomical assessment of coronary arteries without the need for invasive coronary angiography [8,24]. CCTA permits early identification of patients with subclinical but high-risk nonobstructive CAD whose prognosis in terms of future cardiovascular events rate is worse when compared to those without coronary atherosclerosis [25,26,27].

Several anatomical CT scores for the assessment of global atherosclerosis burden at a patient level have been previously described to be associated with cardiovascular prognosis: segment-involvement score (SIS), the segment-stenosis score (SSS), and CT-adapted Leaman score (CT-LeSc) [28,29]. SIS and SSS provide a comprehensive evaluation of atherosclerotic burden by taking into consideration the number of coronary segments involved (SIS) and lumen stenosis degree (SSS).

CT-LeSc (Table 1) is of particular interest as it includes location, severity, and type (calcified vs. noncalcified) of coronary plaque. Of note, patients with nonobstructive CAD and a CT-LeSc more than 5 were reported to have a similar risk of cardiovascular events when compared to patients with obstructive CAD (lumen stenosis > 50%) but a CT-LeSc less than 5 at a mean follow-up of 52 ± 22 months [29]. These findings could be explained by the higher weighting factors attributed to lesions with fibro-lipidic plaque contents and located in more proximal coronary segments, even without significant reduction of coronary lumen (Table 1).

### 2.4. Advanced Atherosclerosis Analysis by CCTA

Advanced coronary plaque analysis by CCTA can be performed by qualitative/semiquantitative methods in the evaluation of a single specific lesion or by more comprehensive methods enabling patient-based atherosclerosis volume quantification (Table 2).

Qualitative/semiquantitative plaque characteristics are lesion-based findings that can be evaluated together or alone in a single coronary plaque and that are defined as outlined in Table 3 [30].

Postprocessing tools recently implemented have resulted in the accurate quantification of coronary artery luminal area, atherosclerotic plaque area, and plaque volume when compared with measurement by IVUS [31]; both low-attenuation plaque (cutoff < 30 HU) and fibro-fatty plaque (cutoff < 130 HU) volumes have been suggested to represent higher-risk atherosclerosis subtypes. It must be underlined that beyond promising results [32], high heart rate, high heart rate variability, and elevated BMI that traditionally may affect the image quality of cardiac CT will reduce the feasibility and accuracy of advanced plaque analysis. Moreover, interscan reproducibility of plaque subtype quantification may be limited by different scan parameters (Kvp and mA) and different coronary lumen contrast attenuation as previously reported [33].

**Figure 4:**
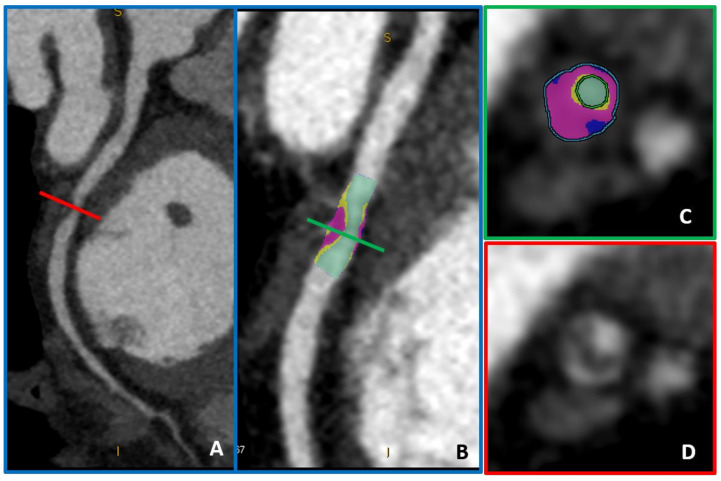
A coronary plaque with moderate lumen stenosis (50–70% stenosis) (panels (**A**,**D**)) can be further analyzed by advanced plaque-volume quantification (panels (**B**,**C**)). In light blue, low-attenuation plaque (HU < 30) is automatically recognized and quantified, while fibro-fatty plaque volume is represented in purple (HU < 130).

Several studies have suggested the important prognostic role of advanced atherosclerosis analysis by CCTA beyond lumen stenosis severity. In 2009, Motoyama et al. demonstrated that acute coronary sindrome (ACS) was independently predicted by postive remodeling (PR) and/or low attenuation plaque (LAP) in a consecutive cohort of patients who underwent CCTA for suspected CAD [22] after 27 ± 10 months. Similar findings have been reported in a selected population of nonobstructive CAD at a longer follow-up of 3.9 ± 2.4 years [27]. In 2018, results from the ICONIC trial [34] showed that the mean number of coronary lesions was similar between patients with and without ACS (3.9 vs. 3.7 lesions per patients, respectively; *p* = 0.40) and patients with ACS did not differ significantly from control subjects in total plaque volume and in calcified plaque volume but had significantly higher fibro-fatty volumes (58.7 ± 85.8 mm^3^ vs. 41.4 ± 62.2 mm^3^; *p* < 0.009). In 2019, results from a SCOT-HEART trial subanalysis showed that adverse coronary plaque characteristics are associated with a three-fold higher risk of coronary heart disease death or nonfatal myocardial infarction. Of interest, in patients with a CACS <100, those with adverse plaque features had an increased risk of coronary events compared to patients without [35]. These results support the fundamental role of low-density/noncalcified plaque-volume quantification for accurate identification of vulnerable plaque that may be missed if only coronary calcium is assessed (Figure 4). Of note, as recently reported from the ICONIC study subanalysis, age and sex may influence plaque subcomponent quantification [36]; more precisely, the calcified plaque volume of a patient increases with age both in female and male subjects, while females have a lower noncalcified plaque volume on a per-patient level at all ages. On the contrary, no age- or sex-related differences were identified in the prevalence of qualitative high-risk plaque features and necrotic core volume. These findings must be taken into consideration to better interpret advanced plaque analysis.

## 3. Current Evidence on CCTA Use among Asymptomatic Patients

Previous data showed the prognostic value of CACS over traditional risk factors in asymptomatic patients, supporting the possible role of CACS in primary prevention (Table 4), even if most of the studies performed included patients with suspected CAD. On the contrary, evidence supporting the prognostic role of CCTA has mainly been obtained in symptomatic patients who underwent CCTA for suspected CAD. Moreover, it should be underlined that both the 2010 AHA/ACC guidelines and the Italian Society of Cardiology recommended against the use of CCTA to rule out CAD in asymptomatic individuals [10,11]. Potential adverse reactions to contrast medium (i.e., allergic reaction and contrast-induced nephropathy), cumulative hazard of radiation exposure, and cost-effectiveness issues supported these recommendations. A recent observational study by Han et al. failed to demonstrate an advantage of CCTA over CACS and clinical evaluation [37]. In 2014, results from the FACTOR-64 trial showed no prognostic benefit of CCTA screening in 900 asymptomatic diabetes patients [38] at a mean follow-up of 4 years; it should be emphasized that no advanced atherosclerosis analysis was performed, and this may have limited benefit of early identification of high-risk atherosclerosis. A subanalysis in the CONFIRM registry suggested that coronary CT angiography may provide incremental prognostic utility over CACS for prediction of mortality and nonfatal myocardial infarction in asymptomatic individuals with moderate CACS values (between 101 and 400) [39] but not in patients with low or very high CACS values. Similarly, in 2015, a study enrolling 2133 asymptomatic patients demonstrated that CCTA enabled the identification of coronary atherosclerosis in 11.4% of the entire population [40]. In 2014, a consecutive cohort of asymptomatic patients was examined with CACS/CCTA, and 71.7% were found to have coronary atherosclerosis with CCTA. While the majority of CACS-zero patients had no coronary stenosis (81%), 12.1% had <50%, 4.6% had intermediate stenosis, and 2.3% had high-grade stenosis by CCTA.

In 2016, Hun-Kang et al. enrolled 591 consecutive asymptomatic patients with type 2 diabetes mellitus who underwent CCTA. The event-free survival rates for a composite endpoint of cardiac death, nonfatal myocardial infarction, and unstable angina requiring hospitalization or late coronary revascularization, at 6 years follow-up, were 99.3% in patients with normal coronary arteries, 96.7% in patients with nonobstructive CAD, and 86.2% in patients with obstructive CAD [43]. Of note, patients without CAD at CCTA have excellent prognosis despite the presence of diabetes as previously suggested [45].

More recently, Halon et al. [44] enrolled 630 asymptomatic diabetic patients who underwent CCTA with advanced atherosclerosis evaluation. Even if the rate of cardiovascular events was low in the entire cohort, high-risk plaque features and elevated fibro-fatty plaque volume (HU < 150) at CCTA were found to be independent predictors of future ACS.

**Figure 5:**
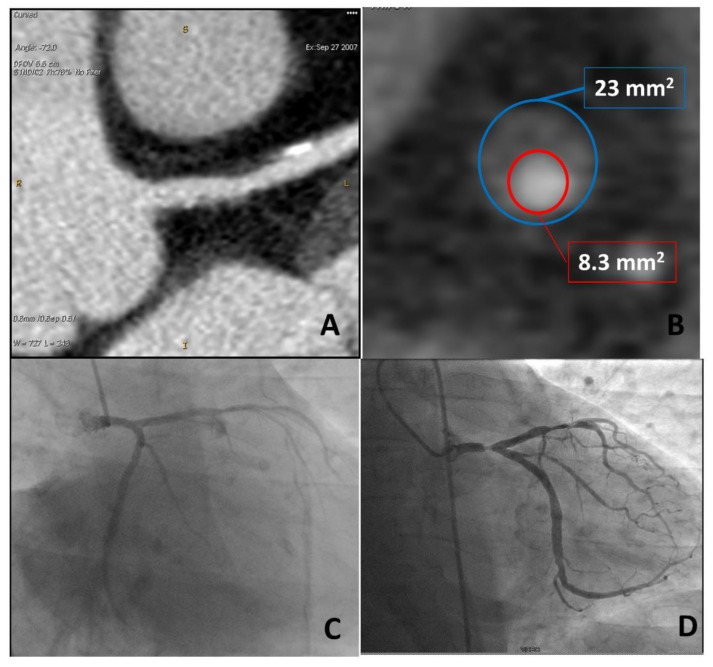
A 51-year-old man underwent CCTA on September 2007 for suspected CAD that showed only moderate lesions (panel (**A**)), with high-risk features on distal left main (positive remodeling and low-attenuation plaque, panel (**B**)). Invasive coronary angiography was performed due to typical anginal symptoms, and no significant diseases were identified (panel (**C**)). In July 2012, the patient’s non-ST elevation myocardial infarction (NSTEMI) and progression to subocclusive disease on the left main was identified as the culprit lesion (panel (**D**)).

Thus, the potential benefit of CCTA over CACS in selected asymptomatic patients may be supported by CCTA’s capability to directly identify location and severity of fibro-lipidic coronary plaque that may be underestimated by CACS but have independent prognostic roles beyond traditional risk factors and coronary lumen stenosis, as recently demonstrated [23,27,28,34,35] (Figure 5). Presently, the systematic combined use of CACS and CCTA in asymptomatic patients is not supported by evidence, but CACS may serve as a gatekeeper for the identification of patients that may merit CCTA, even if asymptomatic. More precisely, low-to-moderate values of CACS may identify patients with the highest probability of having prognostic benefits from CCTA with the identification of subclinical high-risk coronary plaques [39].

## 4. Future Perspectives for Potential Use of CCTA in Primary Prevention

Assuming that CCTA cannot be performed in the entire population and that it is presently not recommended, it is of utmost importance to identify subgroups of patients that may potentially benefit from CCTA as screening tests targeting healthy people. Among others, asymptomatic diabetic patients may benefit from early CCTA, at least for the excellent prognosis if normal coronary arteries are found [33,34,35,36,37,38,39,40,41], even if data from the FACTOR-64 trial failed to demonstrate a prognostic benefit of CCTA [37]. Moreover, several biomarkers like pentraxin, hs-CRP, and hs-Tn-I have been suggested to be associated with high-risk atherosclerosis at CCTA and may help physicians to select healthy but higher-risk subjects [46]. Results from the CAPIRE trial suggested that hscTnT could accurately identify diffuse CAD in patients without acute coronary syndrome or clinical history of CAD and could be considered predictive biomarkers of CAD to identify outlier patients among those with a low-risk-factor burden [46]; moreover, hs-CRP was found to be correlated with high-risk atherosclerosis at CCTA in a recent subanalysis of the CAPIRE trial [47].

Senior athletes are a potential subgroup that may benefit from CCTA even if asymptomatic and without previous cardiovascular disease. Indeed, cardiac CT (CACS, CCTA, or both) may hold the best potential for use in preparticipation screening of older athletes with several cardiovascular risk factors, among which sudden cardiac death is most frequently due to unknown CAD [48].

After appropriate selection of subjects that should undergo CCTA, the absence of therapeutical consequences of nonobstructive but high-risk CAD identification may limit the clinical application of CCTA, as no drug directly acting on atherosclerosis plaque regression or stabilization is currently available for appropriate secondary prevention treatment after early identification of subclinical high-risk coronary atherosclerosis. However, preliminary evidence of atherosclerosis-targeted therapy after CCTA comes from a SCHOT-HEART trial subanalysis: statin and aspirin prescription rate significantly increased after CCTA when compared with a functional test-arm, possibly justifying the prognostic benefit demonstrated in the CCTA arm [35]. Statin therapy has been previously associated with an increase in calcified plaque subcomponents. This effect, called the “statin plaque paradox” with an increase of calcified plaque burden at the expense of fibro-lipidic subcomponents, has been described both at serial IVUS and CT evaluation and was associated with a plaque stabilization process [47,48,49,50]. Of note, the subsequent increase in coronary calcium burden after statin therapy makes CACS a suboptimal tool for the description of statin-related plaque effects. Similarly, in a prospective observational study of 80 patients with recent ACS, colchicine with optimal medical therapy was significantly associated with a reduction in low-attenuation plaque-volume reduction at CCTA, suggesting the potential role of anti-inflammatory therapies in high-risk plaque regression [51]. Similarly, results from the GLAGOV trial suggested a reduction of atheroma volume evaluated at IVUS after treatment with evolocumab [52].

## 5. Limitation to CCTA in Asymptomatic Patients

Despite recent data providing evidence for early identification of subclinical coronary atherosclerosis, it should be underlined that several limitations exist regarding the use of CCTA in asymptomatic patients. One of the main criticisms is the absence of clear-cut evidence supporting the prognostic role of CCTA in this setting and, of most importance, the absence of atherosclerosis-targeted therapy that could be introduced in patients at higher risk; moreover, the risk of potential adverse events associated with the use of intravenous contrast medium (i.e., severe allergic reactions) cannot be underestimated.

## 6. Conclusions

The prognostic role of CACS, even among asymptomatic patients, has been extensively supported by several previous studies and across different ethnic groups [41,53], and its selective use in a primary prevention setting is supported by clinical guidelines [2,19,20,21]. Even if CCTA is currently not recommended in asymptomatic patients, advanced atherosclerosis evaluation by CCTA may have an important role in the future for early and accurate identification of asymptomatic but high-risk patients [42], especially if atherosclerosis-targeted therapies will be introduced into the clinical scenario for secondary prevention. Recent technological advances further reducing radiation dose exposure even below 1 mSv may support the use of CCTA in selected asymptomatic patients [54], but potential adverse reactions to contrast medium are still a matter of concern. Thus, further data are needed to demonstrate the prognostic advantage of early referral to CCTA before this technique can be used routinely in a primary prevention setting.

Overall, these data are shifting the paradigm of coronary artery disease treatment from a lumen- and ischemia-centered view to a more comprehensive approach reflecting the natural history of atherosclerosis progression. As suggested by J. Min in 2016 [55], “the disease severity should be gauged hierarchically: (1) plaque versus no plaque; (2) high-risk plaque features versus non-high-risk plaque features; (3) high-grade stenosis versus non-high-grade stenosis; and finally, (4) ischemia versus no ischemia”, and CCTA appears as the most promising noninvasive tool to serve this new atherosclerosis-centered approach.

## Figures and Tables

**Table 1:** CT-adapted Leaman score.

	Left-Dominance	Right-Dominance	Balanced-Dominance
**Coronary Segments**			
Left main	5	6	5.5
LAD proximal	3.5	3.5	3.5
LAD mid	2.5	2.5	2.5
LAD distal/1st diagonal	1	1	1
2nd diagonal	0.5	0.5	0.5
LCX proximal	1.5	2.5	2
LCX distal	0.5	1.5	1
1st and 2nd marginal	1	1	1
RCA (all segments)	1	0	0.5
PDA	1	Not applicable	0.5
PDA from LCA	Not applicable	1	Not applicable
PL from RCA	0.5	Not applicable	Not applicable
PL from LCA	Not applicable	0.5	0.5
Intermedium	1	1	1
**Stenosis Severity**			
Obstructive CAD	1	1	1
Nonobstructive CAD	0.615	0.615	0.615
**Plaque composition**			
Noncalcified or mixed	1.5	1.5	1.5
Calcified	1	1	1

RCA: right coronary artery, PDA: posterior descending artery, LAD: left anterior descending, LCx: left circumflex, PL: postero-lateral, CAD: coronary artery disease. Modified from de Araújo Gonçalves P et al., *Int J Cardiov Img* 2013. Leaman CT score is obtained by multiplying “coronary segments”, “stenosis severity”, and “plaque composition” factors for each coronary plaque. The Leaman CT score of the patient is obtained by summing all plaque scores.

**Table 2:** Atherosclerosis analysis by cardiac CT.

Patient-Based		Segment-Based	
Conventional Reading	Advanced Analysis	Conventional Reading	Advanced Analysis
Calcium score Segment-involvement score (SIS) Segment-stenosis score (SSS)	CT-adapted Leaman score Plaque-volume quantification	Stenosis degree; Plaque type (calcified/noncalcified)	Remodeling index Low attenuation plaque (LAP) Napkin-ring sign Spotty calcification

**Table 3:** Qualitative high-risk features.

High-Risk Feature	Definition	Example
Remodeling index (RI)	Ratio between lesion plaque area (red circle) and reference lumen area (blue circle). Positive remodeling is defined as RI > 1.1.	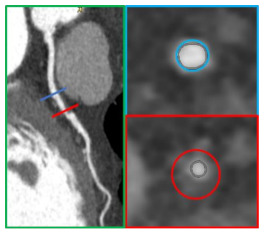
Low-attenuation plaque (LAP)	Presence of any voxel < 30 HU in a coronary plaque.	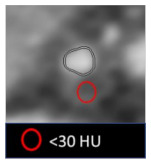
Plaque burden (PB)	Derived by the following formula: (lesion plaque area (red circle)—lesion lumen area (blue circle))/lesion plaque area (red circle).	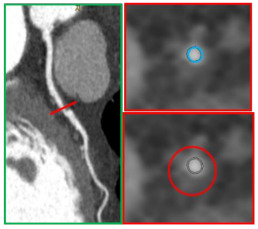
Napkin-ring sign (NRS)	Presence of rim-like thin enhancement (no more than 130 HU) distributed along the outer contour of the vessel and surrounding a fibro-lipidic plaque.	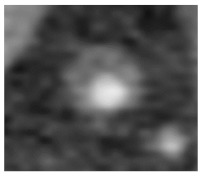
Small spotty calcifications (SC)	Any discrete calcification ≤ 3 mm in length and occupying ≤ 90° arc when viewed on short axis.	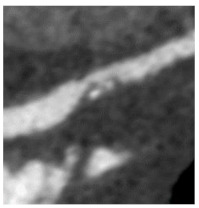

**Table 4:** Main studies reporting prognostic role of CACS and CCTA among asymptomatic patients.

Authors	Publication Year	PopulationNumber	CACS/CCTA	Endpoints	Follow-Up	Main Results	Ref
Kondos et al.	2003	8855	CACS	(1) Death (2) MI (3) Revascularization procedure	37 ± 12 months	CACS provides incremental information vs. conventional CAD risk assessment.	[14]
Taylor et al.	2005	2000	CACS	(1) ACS (2) Sudden cardiac death	3 ± 1.4 years	CACS provides substantial, cost-effective, independent prognostic value incremental coronary risk factors.	[6]
Budoff et al.	2007	25,253	CACS	All-cause mortality	6 ± 3 years	CACS provides independent incremental information in addition to traditional risk factors in the prediction of all-cause mortality.	[15]
Detrano et al.	2008	6722	CACS	(1) MI (2) Death due to coronary heart disease	3.8 years	CACS provides predictive information beyond that provided by standard risk factors.	[41]
Muhlestein et al.	2014	900	CCTA	(1) ACS (2) All causes of death	4 years	Among asymptomatic patients with type 1 or type 2 diabetes, use of CCTA to screen for CAD did not reduce cardiovascular events.	[38]
Cho et al.	2015	3217	CACS and CCTA	(1) MI (2) All causes of death	24 months	CCTA provides incremental prognostic utility for asymptomatic individuals with moderately high CACS, but not for lower or higher CACS.	[39]
Joong Kim et al.	2013	2133	CCTA	(1) ACS (2) Cardiac death (3) Coronary revascularization	29.3 ± 14.9 months	CCTA might have the potential to identify high-risk groups in the selected subjects regarded as a minimal-risk group according toNational Cholesterol Education Program NCEP guidelines.	[40]
Min et al.	2014	27,125	CCTA	(1) ACS (2) Cardiac death (3) Coronary revascularization	2.4 ± 1.1 years	For asymptomatic diabetic individuals, CCTA measures of CAD severity confer incremental risk prediction.	[42]
Kang et al.	2016	591	CCTA	(1) Cardiac deaths (2) Nonfatal MI (3) UA (4) Late coronary revascularizations	6 years	Results suggested long-term prognostic value of coronary CCTA for asymptomatic type 2 diabetes mellitus.	[43]
Halon et al.	2019	630	CCTA	ACS	9.2 years	In asymptomatic patients with type 2 diabetes, CCTA plaque volume, percent low-density plaque content, and mild calcification predicted late plaque events.	[44]

ACS: acute coronary syndrome, MI: myocardial infarction, CCTA: coronary computed tomography angiography, CACS: coronary artery calcium score, UA: unstable angina, CAD: coronary artery disease.

## Data Availability

Not applicable.

## Abbreviation

CCTA	Coronary computed tomography angiography
CACS	Coronary artery calcium score
MACE	Major adverse cardiovascular events
SIS	Segment-involvement score
SSS	Segment-stenosis score
CT-LeSC	CT-adapted Leaman score
IVUS	Intravascular ultrasound
CAD	Coronary artery disease
